# Mitochondrial DNA Haplogroup M7 Confers Disability in a Chinese Aging Population

**DOI:** 10.3389/fgene.2020.577795

**Published:** 2020-10-23

**Authors:** Dayan Sun, Shun Yao, Fei Wu, Wan Deng, Yanyun Ma, Li Jin, Jiucun Wang, Xiaofeng Wang

**Affiliations:** ^1^Ministry of Education Key Laboratory of Contemporary Anthropology, School of Life Sciences, Fudan University, Shanghai, China; ^2^Six-sector Industrial Research Institute, Fudan University, Shanghai, China; ^3^State Key Laboratory of Genetic Engineering and Collaborative Innovation Center for Genetics and Development, School of Life Sciences, Fudan University, Shanghai, China; ^4^Human Phenome Institute, Fudan University, Shanghai, China; ^5^Shanghai Mental Health Center, Shanghai Jiao Tong University School of Medicine, Shanghai, China; ^6^Shanghai Key Laboratory of Psychotic Disorders, Shanghai, China

**Keywords:** mtDNA haplogroup, disability, cybrid, mitochondrial function, aging population

## Abstract

Mitochondrial DNA (mtDNA) haplogroups have been associated with functional impairments (i.e., decreased gait speed and grip strength, frailty), which are risk factors of disability. However, the association between mtDNA haplogroups and ADL disability is still unclear. In this study, we conducted an investigation of 25 mtSNPs defining 17 major mtDNA haplogroups for ADL disability in an aging Chinese population. We found that mtDNA haplogroup M7 was associated with an increased risk of disability (OR = 3.18 [95% CI = 1.29–7.83], *P* = 0.012). The survival rate of the M7 haplogroup group (6.1%) was lower than that of the non-M7 haplogroup group (9.5%) after a 6-year follow-up. In cellular studies, cytoplasmic hybrid (cybrid) cells with the M7 haplogroup showed distinct mitochondrial functions from the M8 haplogroup. Specifically, the respiratory chain complex capacity was significantly lower in M7 haplogroup cybrids than in M8 haplogroup cybrids. Furthermore, an obvious decreased mitochondrial membrane potential and 40% reduced ATP-linked oxygen consumption were found in M7 haplogroup cybrids compared to M8 haplogroup cybrids. Notably, M7 haplogroup cybrids generated more reactive oxygen species (ROS) than M8 haplogroup cybrids. Therefore, the M7 haplogroup may contribute to the risk of disability via altering mitochondrial function to some extent, leading to decreased oxygen consumption, but increased ROS production, which may activate mitochondrial retrograde signaling pathways to impair cellular and tissue function.

## Introduction

Mitochondria are “cellular powerhouses,” which provide > 90% of the cellular ATP through oxidative phosphorylation (OXPHOS). Human mitochondrial DNA (mtDNA) is matrilineal inherited, and encodes 13 essential polypeptides, 2 rRNAs, and 22 tRNAs (Wallace, 2013). mtDNA haplogroups are defined by special mtDNA variants that divide the population into discrete groups, each of which shares a common female ancestor (Sun et al., 2019). Notably, mtDNA variants are closely associated with many age-related diseases, especially common chronic diseases, such as diabetes (Fuku et al., 2007; Kuo et al., 2016), cardiovascular diseases (Sawabe et al., 2011; Qin et al., 2014), and degenerative diseases (van der Walt et al., 2004; Bi et al., 2015). These diseases may contribute to the prevalence of physical function impairments, such as age-related disability.

Characterized by multi-systemic decline, disability refers to the loss of daily life and social activities ability primarily attributable to both physiological and pathological aging, which means decreased physical functioning and cognitive performance, as well as other age-related chronic diseases (Rowe and Kahn, 2000; Gu et al., 2015). Evidence shows that the heritability of disability in populations older than 75 years can be estimated to be 28% (Mikhal’skii et al., 2009). Recently, an analysis of genetic polymorphisms on functional status at very old age (mean age 93.2 years) indicated that for genes in the oxidative stress pathway, several variants were associated with activities of daily living (i.e., TXNRD1-rs7310505) (Dato et al., 2014). Additionally, a number of investigations performed with mtDNA haplogroups and disability-related phenotypes, such as weak grip strength, slow gait speed, and frailty, revealed that mitochondrial haplogroups were associated with phenotypes of disability (Moore et al., 2010; Sun et al., 2018; Erlandson et al., 2020). However, the underlying mechanisms of the mtDNA haplogroups and disability remains unknown. In addition, whether mtDNA haplogroups are associated with ADL disability is also unknown. We thus sought to determine if mtDNA haplogroups contribute to ADL disability by altering mitochondrial function and intracellular mitochondrial signals to some extent. Therefore, we conducted an association study in a Chinese aging population to evaluate the potential correlation between mtDNA haplogroups and the prevalence of disability by ADL assessment. Moreover, we explored the possible effect of mtDNA haplogroups on the pathophysiology of disability by using a cellular model.

## Materials and Methods

### Study Participants

We used data from the longevity arm of the Rugao Longevity and Aging Study (RuLAS). As previously described (Liu et al., 2016), we recruited 463 (103 men and 360 women) participants aged 95–107 years [age (years): mean ± SD = 97.42 ± 2.10] between December 24, 2007 and February 29, 2008. Dates of death were obtained from the Bureau of Civil Affairs of Rugao. Survival time (in months) was calculated from February 2008 to the date of death or of censoring, on April 2014. The Human Ethics Committee of the School of Life Sciences, Fudan University, Shanghai, China, approved the present study. Written informed consent was obtained from all of the participants prior to the study.

### mtSNP Genotype and ADL Score

We selected 25 mtSNPs to define 17 major mitochondrial haplogroups (A, B, B4, B5, D, D4, D5, F, F1, G, G2, M7, M8, M9, M10, M12, and N9) (Supplementary Table S3) found in the Chinese population (Cai et al., 2009). Functional disability was assessed using the Katz Index of activities of daily living (basic activities of daily living, BADL) and the Lawton index of instrumental ADL (IADL) (Katz et al., 1963; Lawton and Brody, 1969). BADL is based on six basic daily tasks: eating, dressing, bathing, indoor transferring, going to the toilet and cleaning oneself afterward. IADL is based on eight instrumental daily task: cooking, doing housework, taking transportation, shopping, washing clothes, making a phone call, managing money and taking medicine. Each task has the following three response alternatives: strongly independent, somewhat dependent, and strongly dependent, with a score of 1, 2, and 3 points, respectively. Lower scores indicated better physical functioning. In this study, disability was defined by Katz Index of ADL, which was modified from the original scale and has been extensively used to evaluate the functional status of older adults. Based on the total summed scores (ranged from 6 to 18), an ADL score > 6 was defined as “disability,” an ADL score = 6 was defined as “normal.”

### Generation of Cell Lines and Culture Conditions

The 143B ρ0 human osteosarcoma cells lacking mtDNA were cultured in high-glucose Dulbecco’s modified Eagle’s medium (DMEM) containing 10% fetal bovine serum (FBS) (Thermo Fisher Scientific, Waltham, MA, United States), 100 μg/mL of pyruvate, and 50 μg/mL of uridine. Trans-mitochondrial cybrids were formed by fusing 143B ρ0 cells and platelets from healthy individuals with corresponding haplogroups, as described previously (King and Attardi, 1989). In this study, cybrids with M7 (*n* = 2) haplogroups and M8 (*n* = 2) haplogroups were constructed. All of the platelets were collected from Taizhou. Cybrids were cultured in high-glucose DMEM containing 10% FBS at 37°C and 5% CO_2_. The morphology of the four cybrids showed no difference (Supplementary Figure S1). Pathogenic mtDNA mutations and cross-contamination were ruled out through Sanger sequencing of the complete mitochondrial genome from all of the cybrids during culture (Supplementary Table S4).

### mtDNA Content Detection

mtDNA content was determined using the 2^(–△^^△^^*CT)*^ method as previously described (Sun et al., 2016). Briefly, genomic DNA was extracted using standard protocols. Quantitative real-time PCR was performed on a QuantStudio 7 Flex Real-Time PCR system (Thermo Fisher Scientific, Waltham, MA, United States) using SYBR Green qPCR Mastermix (Takara Bio, Dalian, China). Primers used were as follows: mt-F: CACCCAAGAACAGGGTTTGT, mt-R: TGG CCATGGGTATGTTGTTAA; 18S DNA-F: TAGAGGGACAAG TGGCGTTC, 18S DNA-R: CGCTGAGCCAGTCAGTGT.

### ATP Measurements

ATP was measured using an ATP measurement kit (Thermo Fisher Scientific, Waltham, MA, United States) according to the manufacturer’s instructions. Briefly, approximately 1 × 10^6^ well-cultured cells were washed with pre-chilled phosphate buffered saline (PBS) buffer, and then boiled in 100 μL lysis buffer for 90 s. Supernatants were retrieved by centrifugation at 10,000 × g for 1 min. ATP content was determined by measuring the luminescence of supernatants mixed with Luciferase Assay buffer using a Spark Multimode Reader (Tecan, Männedorf, Switzerland). ATP luminescence was normalized by protein concentration.

### ROS Measurements

Mitochondrial ROS was measured as described previously (Fang et al., 2015). Briefly, cells were washed in Hank’s buffered salt solution (HBSS), resuspended in DMEM containing 5 μM Mito SOX (Thermo Fisher Scientific, Waltham, MA, United States) and then incubated at 37°C for 5 min. Cells were then washed with HBSS and the fluorescence was recorded using a Spark Multimode Reader (Tecan, Männedorf, Switzerland). ROS fluorescence was normalized based on protein concentration for each sample.

### Oxygen Consumption

Oxygen consumption from intact cells was determined using an Oxygraph-2k instrument (Oroboros, Austria) as described previously (Xu et al., 2017). After recording basal respiration, oligomycin (2.5 μg/mL) (Sigma, MO, United States) was added to measure phosphorylation-coupled respiration.

### Mitochondrial Membrane Potential (MMP) Measurements

MMP was determined using the cationic fluorescent redistribution dye tetramethylrhodamine, methyl ester (TMRM) (Thermo Fisher Scientific, Waltham, MA, United States) as previously described (Sun et al., 2016). Briefly, cells were incubated with a final concentration of 30 nM TMRM at 37°C for 20 min. Cells were then washed with PBS and the fluorescence was recorded using a Spark Multimode Reader (Tecan, Männedorf, Switzerland). MMP fluorescence was normalized to the protein concentration.

### Blue Native PAGE (BN-PAGE), Immunoblotting, and Antibodies

Mitochondrial membrane proteins were extracted with n-dodecyl-D-maltoside (DDM) (Sigma, MO, United States) supplemented with a protease-inhibitor cocktail (Sigma, MO, United States). The detergent/protein ratio was 2.5 g/g. Proteins (60 μg) were then separated by BN-PAGE (4–16% gel) as described previously (Wittig et al., 2006). Western blotting was performed as described elsewhere (Zhuang et al., 2014). OXPHOS complexes were detected with anti-Grim 19 (1:1000, Cat#: ab110240, Abcam, United Kingdom), anti-SDHA (1:1,000, Cat#: ab14715, Abcam, United Kingdom), anti-UQCRC2 (1:1,000, Cat#: ab203832, Abcam, United Kingdom), anti-CO1 (1:1,000, Cat#: ab14705, Abcam, United Kingdom), and anti-ATP5a (1:1,000, Cat#: ab110273, Abcam, United Kingdom) antibodies. Anti-VDAC (1:1,000, Cat#: ab14734, Abcam, United Kingdom) antibody was used as an internal antibody.

### SDS-PAGE, Immunoblotting, and Antibodies

Proteins were extracted with RIPA lysis buffer (CST, Danvers, MA, United States) supplemented with a protease-inhibitor cocktail (Sigma, MO, United States). Proteins were separated by SDS-PAGE, and were blotted with antibodies listed as follows: anti-VDAC (1:1,000, Cat#: ab14734, Abcam, United Kingdom) and anti-GRP75 (1:1,000, Cat#: 14887-1-AP, Proteintech, China), anti-HSP60 (1:1,000, Cat#: 15282-1-AP, Proteintech, China), anti-LONP1 (1:1,000, Cat#: 15440-1-AP, Proteintech, China), and anti-CLPP (1:1,000, Cat#: 15698-1-AP, Proteintech, China). Beta Actin (1:1,000, Cat#: 20536-1-AP, Proteintech, China) antibody was used as an internal antibody.

### Statistical Analysis

The data are presented here as either the mean ± SD or a percentage, with comparisons between different groups being performed using a *t*-test, a Mann–Whitney *U*-test, or a Chi-square test, when appropriate. Haplogroups featuring a frequency > 5% in either the normal participants or disability participants were analyzed in this study. Haplogroups that featured frequencies of < 5% were regarded as “other haplogroups.” Haplogroup D4 was taken as reference class since no study associated with haplogroup D4 and disability related phenotypes, such as decreased gait speed and grip strength, and frailty was reported; in addition, haplogroup D4 was associated with longevity in Chinese (Cai et al., 2009) and Japanese population (Alexe et al., 2007; Bilal et al., 2008). Bonferroni correction indicating a *P* value of < 0.006 (0.05/9) was considered statistically significant when analyzing these 9 haplogroups (with the other haplogroup excluded). The significance of “other haplogroups” was not considered here because the group included multiple mtDNA haplogroups. Logistic regressions were performed to investigate the relationship between haplogroup M7 and disability. Odds ratios (OR) and 95% confidence intervals (CI) were documented. Kaplan–Meier survival analysis was applied to compare the survival of participants with different haplogroups. Cox proportional hazard regression models were used to calculate hazard ratios (HR) and 95% confidence intervals (CI) adjusting for multiple covariates. Statistical analyses were performed using SPSS statistical software 22.0 (IBM Corporation, Armonk, NY). A two-sided *p* < 0.05 was considered to be significant.

## Results

### mtDNA Haplogroup M7 Is Associated With an Increased Risk of Disability

In total, 463 participants aged over 95 years were included in this study (mean age: 97.42 ± 2.10 years). Overall 245 participants (52.9%) were defined as disability (ADL score > 6). The ADL and IADL score of disability group were significantly higher than normal group (11.06 vs. 6.00, *p* < 0.001; 14.55 vs. 10.15, *p* < 0.001, respectively). The survival rate of the disability group (5.5%) was significantly lower than that of the normal group (13.4%) after a 6-year follow-up (*p* = 0.003). Additionally, albumin levels significantly declined in the disability group (41.56 ± 8.86) compared to normal group (43.10 ± 4.23), while the platelets and phosphatase levels were increased in the disability group compared to normal group (Supplementary Table S1).

The distributions of haplogroups M7 in the disability group were significantly higher than those in the normal group (11.0% vs. 3.7%, *p* = 0.002). In addition, we found an increased frequency of M7 (OR = 3.90, 95% CI = 1.42–7.22, *p* = 0.003) using multivariate logistic regression analysis with adjustment for age, sex, and haplogroups (Table 1). Participants with M7 haplogroup had significantly poor performance in activities of daily living, including ADL and IADL, and the percentage of disability was higher than in non-M7 haplogroups (77.1 vs. 50.9%), and the M7 haplogroup groups presented significantly elevated levels of platelets and phosphatase compared to non-M7 haplogroups groups (Table 2). In further analysis, we tested the associations of the M7 haplogroup with disability in 463 participants. The M7 haplogroup was significantly associated with disability (OR = 3.18, 95% CI = 1.29–7.83, *P* = 0.012) after adjusting for sex, age, marriage, smoking habits, drinking habits, BMI, SBP, DBP, hemoglobin, platelets, white blood cell, albumin, phosphatase, UA, CHOL, TG, LDL, and HDL (Supplementary Table S2). After a 6-year follow-up (median follow-up times: 30 months), 410 participants died. Kaplan–Meier survival analysis showed that the survival rate of the M7 haplogroup (6.1%) was lower than that of the non-M7 haplogroup (9.5%) after a 6-year follow-up, LogRank *p* = 0.188 (Supplementary Figure S3). Meanwhile, Cox proportional hazard regression models were used to calculate HR (HR = 1.27, 95% CI = 0.88–1.83, *p* = 0.196) after adjusting for sex and age. Albeit no significance was observed due to the small samples of M7 haplogroup, we still noticed that after 2 years, the survival rate difference between M7 haplogroup and non-M7 haplogroups was considerable.

**TABLE 1:** Multivariate logistic regression analysis of mitochondrial haplogroups associated with disability with adjustment for age, sex, and haplogroup.

Haplogroups	Normal (*n* = 218)	Disability (*n* = 245)	OR (95% CI)	*p*-value
A	17 (7.8%)	18 (7.3%)	1.26 (0.57–2.79)	0.558
B	32 (14.7%)	44 (18.0%)	1.63 (0.87–3.05)	0.125
D4	47 (21.6%)	41 (16.7%)	1.0	
D5	11 (5.0%)	19 (7.8%)	2.18 (0.91–5.19)	0.077
F	37 (17.0%)	40 (16.3%)	1.27 (0.68–2.36)	0.441
G	11 (5.0%)	10 (4.1%)	1.07 (0.41–2.81)	0.886
M7	8 (3.7%)	27 (11.0%)	3.90 (1.59–9.56)	0.003
M8	27 (12.4%)	20 (8.2%)	0.89 (0.43–1.84)	0.769
N9	11 (5.0%)	11 (4.5%)	1.14 (0.45–2.93)	0.772
Others*	17 (7.7%)	15 (6.1%)	–	–

Values in parentheses are the percentage of samples. CI, confidence interval; OR, odds ratio. *represents haplogroup with frequencies <5% in normal and disability group. P < 0.005 (0.05/9), adjusted p-value with Bonferroni correction while 9 haplogroups studied in this study.

**TABLE 2:** Analysis of mtDNA haplogroup M7 and disability.

	Non-M7	M7	*p-*value
	(*n* = 428)	(*n* = 35)	
Age	97.41 ± 2.12	97.63 ± 1.81	0.548
Gender (%)			0.302
Male	97 (22.7%)	6 (17.1%)	
Female	331 (77.3%)	29 (82.9%)	
Marriage status			0.704
Married	22 (5.1%)	1 (2.9%)	
Widowed	402 (93.9%)	34 (97.1%)	
Unmarried	4 (0.9%)	0 (0.0%)	
Smoking habits			0.044
Smoked	38 (8.9%)	0 (0.0%)	
Never smoked	390 (91.1%)	35 (100.0%)	
Drinking habits			0.32
Drink	146 (34.1%)	10 (28.6%)	
Never	282 (65.9%)	25 (71.4%)	
Height (cm)	153.54 ± 11.29	153.79 ± 6.98	0.895
Weight (kg)	51.43 ± 11.45	47.91 ± 7.43	0.079
BMI (kg/m2)	21.63 ± 4.23	20.27 ± 2.94	0.067
SBP (mmHg)	136.93 ± 22.95	134.11 ± 18.42	0.481
DBP (mmHg)	80.07 ± 11.05	79.27 ± 10.66	0.68
GLU (mmol/L)	4.97 ± 1.35	4.67 ± 0.89	0.316
Disability	50.9%	77.1%	0.002
ADL	8.56 ± 3.60	10.34 ± 3.66	0.006
IADL	12.38 ± 3.64	13.89 ± 3.20	0.018
Hemoglobin (g/L)	128.50 ± 19.99	127.88 ± 21.57	0.865
Platelets(10^^9^/L)	163.91 ± 67.93	190.61 ± 60.88	0.029
White blood cell (10^^9^/L)	5.39 ± 1.62	5.83 ± 2.21	0.142
Albumin (g/L)	42.30 ± 4.65	41.96 ± 4.58	0.691
Protein (g/L)	70.10 ± 6.58	70.66 ± 6.25	0.637
Phosphatase (IU/L)	85.38 ± 25.43	97.45 ± 28.78	0.01
Creatinine (μmol/L)	65.42 ± 20.08	65.67 ± 18.02	0.956
UA (μmol/L)	280.85 ± 94.53	290.36 ± 87.45	0.576
CHOL (mmol/L)	4.77 ± 0.94	4.96 ± 1.15	0.251
TG (mmol/L)	1.10 ± 0.51	1.10 ± 0.41	0.999
LDL (mmol/L)	2.49 ± 0.69	2.56 ± 0.71	0.56
HDL (mmol/L)	1.37 ± 0.33	1.15 ± 0.42	0.186
Status of survival (%)			0.389
Survival	40 (9.5%)	2 (6.1%)	
Death	379 (90.5%)	31 (93.9%)	

**Values in parentheses are the percentage of samples. BMI, body mass index; SBP, systolic blood pressure; DBP, diastolic blood pressure; GLU, glucose in urine; UA, uric acid; CHOL, cholesterol; TG, triglyceride; LDL, low density lipoprotein; HDL, high density lipoprotein. P-value: Chi-square test was used in the table.**

### M7 Cybrids Exhibit Lower Respiration Chain Complex (RCC) Capacity Than M8 Cybrids

**FIGURE 1:**
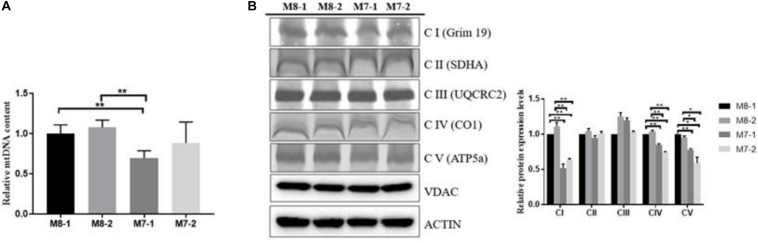
M7 cybrids exhibit decreased respiratory chain complex (RCC) amount relative to M8 cybrids. **(A)** mtDNA content of M8 and M7 cybrids. Relative mtDNA content were normalized to M8-1 cybrid. **(B)** Whole-cell extract of mitochondrial respiratory complexes from M8 and M7 cybrids solubilized with dodecyl maltoside (DDM) at a ratio of 2.5 g of detergent/g protein and then subjected to blue native PAGE (BN- PAGE)/immunoblot (IB) analysis. Complexes I, II, III, IV, and V were immunoblotted with anti-Grim19, SDHA, UQCRC2, COX1, and ATP5a antibodies, respectively; VDAC was used as a total-protein loading control. Protein levels were normalized to M8-1 cybrid. Data are presented as the mean ± SD from at least 3 independent tests per experiment. **P < 0.01.

To uncover the biological mechanism of the M7 haplogroup and disability, we generated cytoplasmic hybrid (cybrid) cell lines containing M7 or M8 haplogroups with an osteosarcoma 143B nuclear background. To analyze the effect of mtDNA haplogroups on the regulation of mitochondrial RCC, we first determined the mtDNA content in M7 and M8 cybrids. The mtDNA content in M8 cybrids was 20–30% higher than that in M7-1 cybrid, and a decreased tendency was also observed in M7-2 cybrid (Figure 1A). The lower mtDNA content measured in M7 cybrids was not because of the reduced number of mitochondria (Figure 1B). Next, an examination of the whole complex amount by BN-PAGE revealed that complexes I, IV, and V abundant were significantly higher in M8 cybrids than in M7 cybrids (Figure 1B). Collectively, our results demonstrated that M7 cybrids exhibited lower RCC capacity than M8 cybrids.

### Mitochondrial Function Is Lower in M7 Cybrids Than M8 Cybrids

**FIGURE 2:**
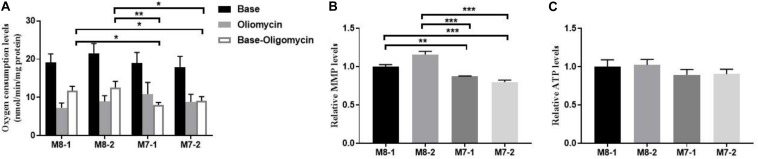
Mitochondrial function is lower in M7 cybrids than M8 cybrids. **(A)** Mitochondrial respiratory capacities were determined in M8 and M7 cybrids. Oligomycin (2.5 μg/mL) was added for the measurement of uncoupled mitochondrial respiration. OXPHOS coupling respiration was calculated by subtracting the uncoupled component value from the total endogenous respiration value. **(B)** MMP was determined in M8 and M7 cybrids treated with 30 nM tetramethylrhodamine (TMRM) for 20 min. Relative MMP levels were normalized to M8-1 cybrid. **(C)** ATP was determined in M8 and M7 cybrids with boiled cells. Relative ATP levels were normalized to M8-1 cybrid. Data are shown as the mean ± SD from at least three independent tests per experiment. *P < 0.05; **P < 0.01, ***P < 0.001.

Next, we measured mitochondrial respiratory profiles of our cybrids using an Oroboros O2k instrument. A respiration assay of the cybrids showed higher total oxygen consumption levels in M7 cybrids than in M8 cybrids. M7 cybrids also presented about a 40% decline in mitochondrial ATP-linked oxygen consumption than M8 cybrids (Figure 2A). Furthermore, M7 cybrids displayed significantly decreased MMP levels (Figure 2B), and a 10% reduction in the ATP generation tendency but without significance (Figure 2C). Taken together, these results indicated that M7 cybrids exhibited diminished mitochondrial function relative to M8 cybrids.

### Mitochondrial Oxidative Stress Is Activated in M7 Cybrids Than M8 Cybrids

**FIGURE 3:**
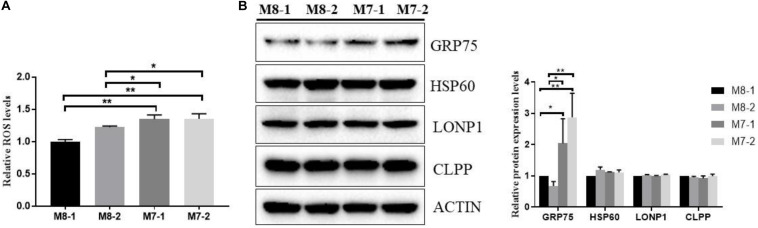
Mitochondrial oxidative stress is activated in M7 cybrids. **(A)** Mitochondrial ROS was determined in M8 and M7 cybrids by MitoSOX. Relative mitochondrial ROS levels were normalized to M8-1 cybrid. **(B)** Representative Western blot of mitochondrial quality control proteins, including GRP75, HSP60, LONP1, and CLPP, from whole-cell extracts of M8 and M7 cybrids. ACTIN was used as a loading control. Relative protein levels were normalized to M8-1 cybrid. Data are presented as the mean ± SD from at least 3 independent tests per experiment. *P < 0.05; **P < 0.01.

Mitochondrial dysfunction can activate mitochondrial signaling mediators to play a critical role in cellular physiology, such as ROS generation (Chae et al., 2013). As shown in Figure 3A, M7 cybrids generated approximately 20–30% enhanced ROS levels compared to M8 cybrids. Together with this, the detection of a significantly higher level of the mitochondrial quality control protein GRP75, which plays an important role in cell proliferation, stress response, and maintenance of the mitochondria, was observed in M7 cybrids relative to M8 cybrids. This supported the notion that M7 cybrids exhibit increased mitochondrial unfolded protein response (mtUPR) as compared to M8 cybrids (Figure 3B). This also suggested that M7 cybrids need to cope with increased ROS stress. However, we found that other mtUPR proteins did not differ between the M7 and M8 cybrids, and thus, the mtUPR level in M7 cybrids may be limited.

## Discussion

In the present study, we first investigated a population study to explore the effects of mtDNA haplogroups on disability in a Chinese aging population. We found that the mtDNA haplogroup M7 was associated with an increased risk of disability independent of several blood biomarkers and common diseases. Furthermore, the survival rate of the M7 haplogroup group was lower than that of M8 haplogroup group at 6 years’ follow-up. Moreover, we observed decreased mitochondrial biogenesis and OXPHOS dysfunction, while elevated ROS production in M7 haplogroup cybrids compared to M8 haplogroup cybrids using a trans-mitochondrial cellular model.

The non-synonymous mutations of haplogroups M7 cybrids located on subunits of complex I, III, IV, and V, such as G4048A (ND1, p. Asp248Asn), C14766T (Cytb, p. Thr7Ile), G7853A (COII, p. Val90Ile), A8701G (ATPase, p. Thr59Ala). Complex I is the largest and first complex of the mitochondrial respiration chain, and oxygen consumption happens in association with complex IV, and ATP finally generates at complex V. Mutations in these complexes lead to serious mtDNA-related diseases, such as mitochondrial encephalomyopathy, Leigh’s syndrome, and Leber hereditary optic neuropathy (LHON) (Schon et al., 2012). Although the mutations in haplogroup M7 have not been reported with diseases, we cannot deny that these mutations may be functional to some extent. In our study, we found that mitochondrial complex I, IV, and V capacities were decreased in M7 haplogroup cybrids compared with M8 haplogroup cybrids. Possible explanations might include that these mutations alter complex structure and assembly to further affect complex capacity and function.

Accumulating evidences demonstrated that mtDNA haplogroups were correlated with longevity. One study clarified that the mtDNA haplogroup frequency distribution between centenarians and younger individuals were obviously different. And the haplogroup J was significantly associated with centenarians in Italy (De Benedictis et al., 1999). Another study also demonstrated that mtDNA haplogroup J increased the individual chance to attain longevity in northern Italians, Northern Irish and Finns (Dato et al., 2004). In addition, mtDNA haplogroup D4a contributed to Japanese semi-supercentenarians (aged above 105 years) (Bilal et al., 2008). Notably, mitochondrial haplogroups may play roles in common chronic diseases with age, such as the role of the N9a haplogroup in diabetes, the B5 haplogroup involvement in Alzheimer’s disease (AD), or the role of G haplogroup in osteoarthritis (Bi et al., 2015; Fang et al., 2016, 2018). Previous studies have found that the mitochondrial M7 haplogroup is predisposed to susceptibility to common chronic diseases such as chronic obstructive pulmonary disease, coronary atherosclerosis, lung cancer, and hepatocellular carcinoma (Sawabe et al., 2011; Zheng et al., 2012a,b; Chen et al., 2017). Notably, evidence has shown that haplogroup M7a is closely associated with Japanese Parkinson’s disease (PD). PD patients have many phenotypes, such as resting tremors, muscle rigidity, gait and posture disorders, and motor retardation. This directly increases the possibility of disability (Takasaki, 2008). A sub-haplogroup of M7b, M7b1′2, is associated with the most extensively studied mitochondrial disease, LHON (Ji et al., 2008). The link of M7 to disability may to some extent be mediated by the effects of M7 on common chronic diseases, which impair multiple organs and physical function.

Although data addressing the relationship between mtDNA haplogroups and disability are scarce, some evidences showed that mtDNA haplogroups were associated with several disability-related phenotypes, including frailty, weak grip, slow gait, and skeletal muscle fatigability (Moore et al., 2010; Sun et al., 2018; Erlandson et al., 2020). Mitochondrial haplogroup H is independently associated with weak grip and frailty among those > 50 years old with HIV (Erlandson et al., 2020). mtDNA T204C is associated with a greater likelihood of frailty and lower muscle strength (Moore et al., 2010). Interestingly, studies of elite athletes with mtDNA variations also demonstrated that mitochondria are associated with physical performance, especially skeletal muscle. For example, mitochondrial haplogroup T is negatively associated with the status of elite endurance athletes in Spain (Castro et al., 2007). Similar studies have also been conducted in other populations, such as Kenyan, Japanese, and Finnish (Niemi and Majamaa, 2005; Scott et al., 2009; Mikami et al., 2011). All of these studies revealed that mitochondrial inheritance is influential in variations in physical performance, and may eventually contribute to disability phenotypes.

The cytoplasmic hybrid (cybrid) technique is widely used for studying how mtDNA variants influence mitochondrial functions in cellular physiological conditions. For example, cybrids with the mitochondrial haplogroup G related to osteoarthritis exhibit altered mitochondrial complexes I and III capacity (Fang et al., 2016). As shown in our results, M7 cybrids exhibited significantly lower respiration chain complexes I, IV, and V amount than M8 cybrids. Additionally, cybrids with diabetes associations, such as the N9a haplogroup, present as impaired mitochondrial function, including decreased ATP content, MMP level, and oxygen consumption (Fang et al., 2018). Consistent with this, M7 cybrids displayed diminished mitochondrial function as compared to M8 cybrids, including ATP-linked respiration and MMP level. Furthermore, N9a haplogroup cybrids exhibited increased ROS production and mild oxidative-stress response (Fang et al., 2018). In our study, superabundant ROS level in M7 cybrids enhanced mitochondrial quality control protein expression, yet, a shortage of oxidative-stress response was observed in M7 cybrids because only GRP 75 was modified, with no change in other quality control protein expression. Taken together, the M7 haplogroup exhibited decreased mitochondrial complex capacity, oxygen consumption, and MMP level, while increased ROS production, which may contribute to the risk of disability to some extent.

It is noteworthy that enhanced mitochondrial ROS production has been implicated as one of the important reasons for the deterioration of DNA, proteins, and lipids in cells (Wallace, 2005). Several studies have revealed that enhanced ROS production induces mitochondrial respiration chain protein dysfunction and apoptosis in aging skeletal muscle (Short et al., 2005; Chabi et al., 2008; Gomes et al., 2017). Recently, a study group performed a longitudinal and deep multiomic profiling of 106 healthy individuals from 29 to 75 years of age and examined comprehensive measurements, including transcript levels, protein levels, metabolites, cytokines, microbes, and clinical laboratory values, and correlated all of them with age. Their data showed that increased oxidative stress response and abnormal ROS production impaired immunity pathways, liver and kidney function, metabolism, and inflammation with age (Ahadi et al., 2020). Notably, mitochondrial dysfunction may alter signals, such as ROS, AMP, and Ca^2+^, which could further change the activities of various nuclear transcription factors, and affect cellular processes, including proliferation, apoptosis, and metabolism (Chae et al., 2013; Picard et al., 2014). The mitochondrial retrograde signaling pathway has been shown to be involved in many common chronic diseases, such as diabetes, osteoarthritis, and AD. For instance, ROS-mediated ERK1/2 signaling increases the incidence of N9a haplogroup-related type II diabetes in Chinese populations (Fang et al., 2018). Moreover, osteoarthritis-associated haplogroup G cybrids had a lower ROS production and cell viability than haplogroup B4 cybrids under hypoxia, although with higher complex I and III activity levels, and ATP generation (Fang et al., 2016). The important mechanism was a shift in the metabolic profile from glycolysis to OXPHOS and activation of osteoarthritis-related signaling pathways Additionally, mtDNA haplogroup B5 conferred genetic susceptibility to AD through decreased mitochondrial function and elevated ROS in Han Chinese (Bi et al., 2015).

Thus, we suggest that the mtDNA haplogroup M7 contributes to physical function-related phenotypes and disability via mitochondrial dysfunction to some extent, with decreased mitochondrial complex capacity and oxygen consumption. Notably, the elevated ROS levels in the M7 haplogroup may activate mitochondrial retrograde signaling pathways to further affect cellular function and disability. This potential mechanism needs further research.

The strengths of our study include the population-based approach, the reasonably large sample size of extreme longevity individuals, and the similar genetic environment, which ensures the homogeneity of our study population. This made it feasible to collect disability phenotypes based on extreme longevity (≥ 95 years old). However, the survival analysis only presented a lower tendency in M7 haplogroup participants than those non- M7 haplogroup participants due to the small samples of M7 haplogroup, this association needs to be validated in other independent samples. Further molecular mechanisms also need to be studied based on our preliminary cellular functional results. In addition, it is difficult for these extremely aged participants to accomplish physical performance measurements (i.e., gait speed and hand grip strength) accurately. Therefore, we could not conduct association analyses between mtDNA haplogroups and these disability-related phenotypes to validate our population findings.

## Conclusion

In summary, we first identified a positive association between the mitochondrial M7 haplogroup and disability in an aging population. Moreover, we demonstrated that the M7 haplogroup exhibits declined mitochondrial biogenesis and function, while enhanced ROS production, which could be attributed to weak physical performance, disability, and even mortality. Our findings provide a primary but crucial role, to some extent, for further molecular mechanism exploration in the field of disability.

## Data Availability Statement

The datasets presented in this study can be found in online repositories. The names of the repository/repositories and accession number(s) can be found in the article/ Supplementary Material.

## Ethics Statement

The studies involving human participants were reviewed and approved by The Human Ethics Committee of the School of Life Sciences, Fudan University, Shanghai, China. The patients/participants provided their written informed consent to participate in this study.

## Author Contributions

SY and WD collected the questionnaires. DS and FW performed the experiments. SY and DS analyzed the data. YM provided plasma for the cellular model. XW, JW, and LJ designed the study. DS wrote the manuscript. XW and JW edited the manuscript. All authors were involved in final approval of the submitted and published versions.

## Conflict of Interest

The authors declare that the research was conducted in the absence of any commercial or financial relationships that could be construed as a potential conflict of interest.

## Supplementary Material

Supplementary Figure 1 ADL score of the participants in this study. Four hundred and sixty three participants were involved in this study. Two hundred and eighteen participants were healthy controls, and Two hundred forty five participants were disability cases.

Supplementary Figure 2 Morphometric analysis of M7 cybrids and M8 cybrids. Morphology of M8 and M7 cybrids in their best growth conditions. The scale bar is 200 μm.

Supplementary Figure 3 Kaplan-Meier survival analysis of M7 and non-M7 haplogroup groups. Kaplan–Meier survival analysis shows the survival rate of the M7 haplogroup group and non-M7 haplogroup groups after a 6-year follow-up. LogRank = 0.188.

Supplementary Table 1 Distribution of basic indicators in different disability groups.

Supplementary Table 2 Multivariate regression analysis of mtDNA haplogroup M7 and disability.

Supplementary Table 3 Decision mtSNPs for haplogroup assignment.

Supplementary Table 4 Analysis of whole mitochondrial genome.
