# Supplementary material for: Mitochondrial DNA Haplogroup M7 Confers Disability in a Chinese Aging Population
Source: Front Genet. 2020 Oct 23;11:577795. doi: 10.3389/fgene.2020.577795 (PMC7645148; doi:10.3389/fgene.2020.577795)
Supplement: Supplementary Figure 1 — ADL score of the participants in this study. Four hundred and sixty three participants were involved in this study. Two hundred and eighteen participants were healthy controls, and Two hundred forty five participants were disability cases. [file Data_Sheet_1.docx]

**Supplementary figures**

**Figure S1. ADL score of the participants in this study.**

463 participants were involved in this study. 218 participants were healthy controls, and 245 participants were disability cases.


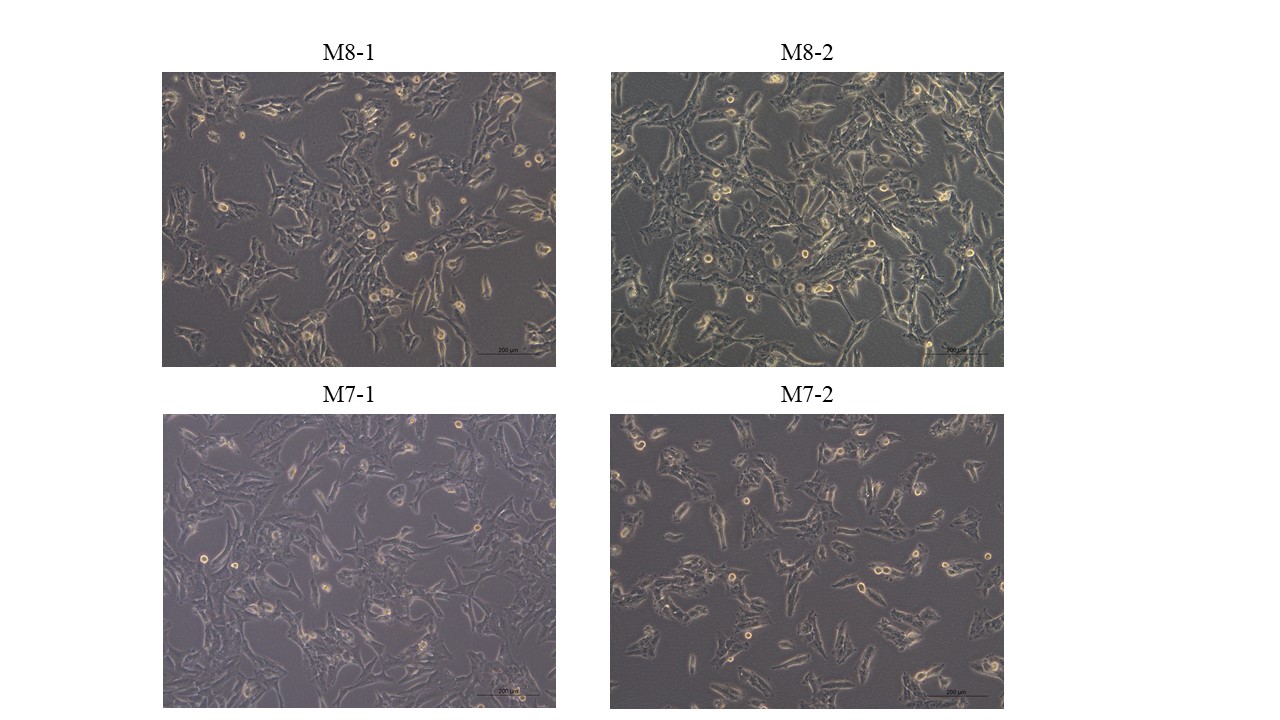


**Figure S2. Morphometric analysis of M7 cybrids and M8 cybrids.**

Morphology of M8 and M7 cybrids in their best growth conditions. The scale bar is 200 µm.


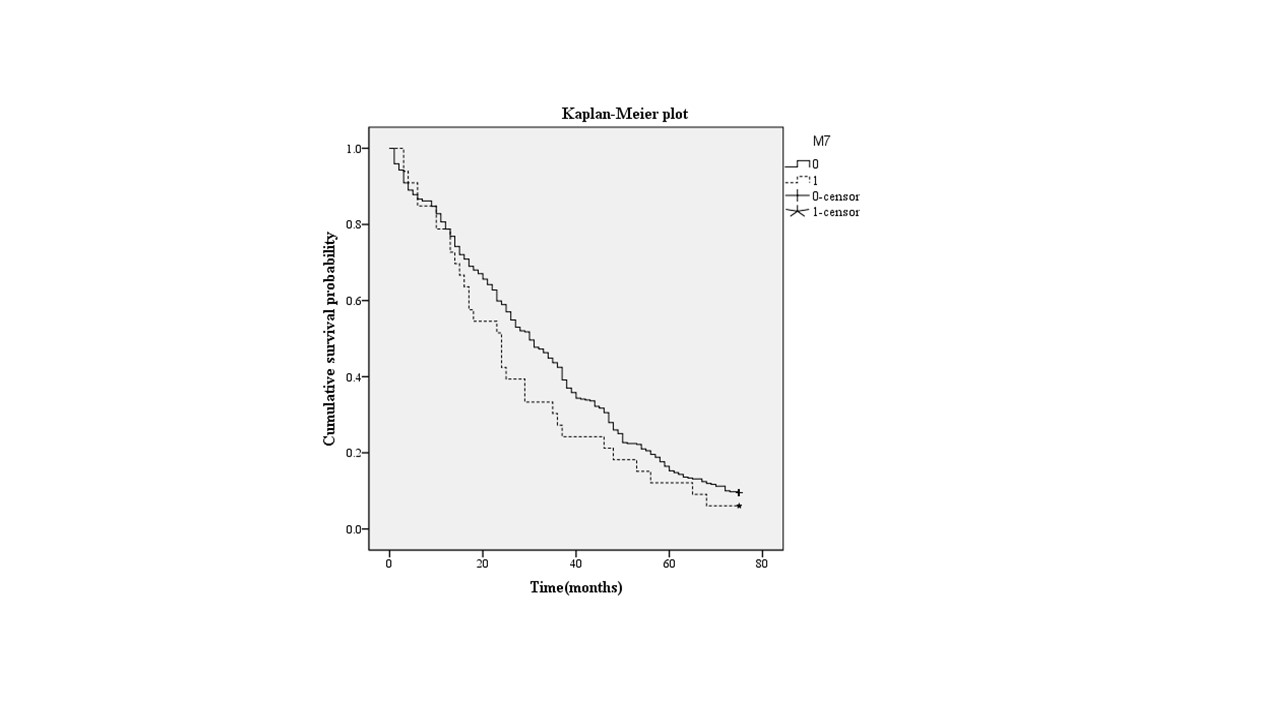


**Figure S3. Kaplan-Meier survival analysis of M7 and non-M7 haplogroup groups.**

Kaplan–Meier survival analysis shows the survival rate of the M7 haplogroup group and non-M7 haplogroup groups after a six-year follow-up. LogRank =0.188.

**Table S1 Distribution of basic indicators in different disability groups**

|  | Normal | Disability | All | p-value |
| --- | --- | --- | --- | --- |
|  | (n = 218) | (n = 245) | (n = 463) |  |
| Age | 97.25 ± 2.02 | 97.58 ± 2.16 | 97.42 ± 2.10 | 0.095 |
| Gender (%) |  |  |  | 0.098 |
| Male | 55 (25.2%) | 48 (19.5%) | 103 (22.2%) |  |
| Female | 163 (74.8%) | 197 (80.5%) | 360 (77.8%) |  |
| Marriage status |  |  |  | 0.875 |
| Married | 12 (5.5%) | 11 (4.5%) | 23 (5.0%) |  |
| Widowed | 204 (93.6%) | 232 (94.7%) | 436 (94.2%) |  |
| Unmarried | 2 (0.9%) | 2 (0.8%) | 4 (0.9%) |  |
| Smoking habits |  |  |  | 0.292 |
| Smoked | 20 (9.2%) | 18 (7.3%) | 38 (8.2%) |  |
| Never smoked | 198 (90.8%) | 227 (92.7%) | 425 (91.8%) |  |
| Drinking habits |  |  |  | 0.213 |
| Drink | 78 (35.8%) | 78 (31.8%) | 156 (33.7%) |  |
| Never | 140 (64.2%0 | 167 (68.2%0 | 307 (66.3%) |  |
| Height (cm) | 153.47 ± 10.16 | 153.63 ± 11.78 | 153.55 ± 11.03 | 0.873 |
| Weight (kg) | 52.01 ± 10.88 | 50.40 ± 11.53 | 51.17 ± 11.24 | 0.125 |
| BMI (kg/m2) | 21.91 ± 4.19 | 21.18 ± 4.12 | 21.53 ± 4.17 | 0.062 |
| SBP (mmHg) | 138.93 ± 22.62 | 134.72 ± 22.52 | 136.71 ± 22.64 | 0.046 |
| DBP (mmHg) | 80.48 ± 11.05 | 79.59 ± 10.99 | 80.01 ± 11.02 | 0.382 |
| Hypertension | 111 (50.9%) | 101 (41.2%) | 212 (45.8%) | 0.023 |
| GLU (mmol/L) | 4.87 ± 1.23 | 5.01 ± 1.40 | 4.95 ± 1.32 | 0.381 |
| ADL | 6.00 ± 0.00 | 11.06 ± 3.62 | 8.33 ± 3.66 | <0.001 |
| IADL | 10.15 ± 3.00 | 14.55 ± 2.80 | 12.47 ± 3.64 | <0.001 |
| Hemoglobin (g/L) | 128.44 ± 19.42 | 128.47 ± 20.68 | 128.45 ± 20.09 | 0.987 |
| Platelets (10^^9^/L) | 158.65 ± 59.16 | 172.16 ± 73.94 | 165.87 ± 67.73 | 0.035 |
| White blood cell (10^^9^/L) | 5.40 ± 1.60 | 5.44 ± 1.74 | 5.42 ± 1.67 | 0.79 |
| Albumin (g/L) | 43.10 ± 4.23 | 41.56 ± 8.86 | 42.27 ± 4.64 | <0.001 |
| Protein (g/L) | 70.61 ± 6.54 | 69.74 ± 6.55 | 70.15 ± 6.55 | 0.159 |
| Phosphatase (IU/L) | 82.65 ± 23.23 | 89.40 ± 27.59 | 86.26 ± 25.85 | 0.006 |
| Creatinine (µmol/L) | 67.92 ± 20.33 | 63.34 ± 19.36 | 65.43 ± 19.91 | 0.052 |
| UA (µmol/L) | 286.97 ± 94.05 | 276.78 ± 92.83 | 281.55 ± 93.97 | 0.253 |
| CHOL (mmol/L) | 4.79 ± 0.95 | 4.77 ± 0.96 | 4.78 ± 0.96 | 0.813 |
| TG (mmol/L) | 1.07 ± 0.47 | 1.12 ± 0.52 | 1.10 ± 0.50 | 0.273 |
| LDL (mmol/L) | 2.50 ± 0.70 | 2.49 ± 0.68 | 2.50 ± 0.69 | 0.923 |
| HDL (mmol/L) | 1.40 ± 0.33 | 1.35 ± 0.34 | 1.37 ± 0.34 | 0.152 |
| Status of survival (%) |  |  |  | 0.003 |
| Survival | 29 (13.4%) | 13 (5.5%) | 42 (9.3%) |  |
| Death | 187 (86.6%) | 223 (94.5%) | 410 (90.7%) |  |

Values in parentheses are the percentage of samples. BMI: body mass index; SBP: systolic blood pressure; DBP: diastolic blood pressure; GLU: glucose in urine; UA: uric acid; CHOL: cholesterol; TG: triglyceride; LDL: low density lipoprotein; HDL: high density lipoprotein. P-value: Chi-square test was used in the table.

**Table S2 Multivariate regression analysis of mtDNA haplogroup M7 and disability**

|  | Model 1 | Model 2 | Model 3 |
| --- | --- | --- | --- |
|  | OR (95% CI) | OR (95% CI) | OR (95% CI) |
| Non-M7 | Ref | ref | ref |
| M7 | 3.28 (1.46 - 7.39) | 3.20 (1.42 - 7.22) | 3.18 (1.29 - 7.83) |
| p-value | 0.004 | 0.005 | 0.012 |

CI, confidence interval; OR, odds ratio.

Model 1: Crude model;

Model 2: Age, Sex;

Model 3: Age, Sex, Marriage, Smoking habits, Drinking habits, Hemoglobin, BMI, SBP, DBP, Platelets, White blood cell, Albumin, Phosphatase, UA, CHOL, TG, LDL, HDL.

**Table S3 Decision mtSNPs for haplogroup assignment**

|  |  |
| --- | --- |
| Haplogroup | Key multiplex SNP variants |
| A | 1736G 10873T |
| B | 9bp- deletion 10873T |
| B4 | T16217C 9bp- deletion 10873T |
| B5 | 8584A 9bp- deletion 10873T |
| D | 5178A 10400T |
| D4 | 3010A 5178A 10400T |
| D5 | 1107C 5178A 10400T |
| F | 3970T 12705C 10873T |
| F1 | 12406A 3970T 12705C 10873T |
| G | 4833G 14569A 10400T |
| G2 | 7600A 4833G 14569A 10400T |
| M7 | 6455T 9824C 10400T |
| M8 | 7196A 10400T |
| M9 | 4491A 3705G 10400T |
| M10 | 10646A 10400T |
| M12 | 14569A 10400T |
| N9 | 5417A 10873T |

**Table S4 Analysis of whole mitochondrial genome**

| position | Gene | rCRS base | mutation | | | | AA change | mtDNA database** |
| --- | --- | --- | --- | --- | --- | --- | --- | --- |
|  |  |  | M8-1 | M8-2 | M7-1 | M7-2 |  |  |
| 73 | D-loop | A | G | G | G | G | no | polymorphic site |
| 150 | D-loop | C |  |  | T | T | no | polymorphic site |
| 152 | D-loop | T | C |  |  |  | no | polymorphic site |
| 199 | D-loop | T |  |  | C | C | no | polymorphic site |
| 204 | D-loop | T |  |  | C | C | no | polymorphic site |
| 263 | D-loop | A | G | G | G | G | no | polymorphic site |
| 489 | D-loop | T | C | C | C | C | no | polymorphic site |
| 509 | D-loop | C |  |  | T |  | no | polymorphic site |
| 750 | 12S rRNA | A | G | G | G | G | no | polymorphic site |
| 1438 | 12S rRNA | A | G | G | G | G | no | polymorphic site |
| 2706 | 16S rRNA | A | G | G | G | G | no | polymorphic site |
| 2835 | 16S rRNA | C |  | T |  |  | no | polymorphic site |
| 3483 | ND1 | G |  |  |  | A | no | polymorphic site |
| 4048 | ND1 | G |  |  | A | A | Asp > Asn | polymorphic site |
| 4071 | ND1 | C |  |  | T | T | no | polymorphic site |
| 4164 | ND1 | A |  |  | G | G | no | polymorphic site |
| 4670 | ND2 | C |  | T |  |  | no | polymorphic site |
| 4715 | ND2 | A | G | G |  |  | no | polymorphic site |
| 4769 | ND2 | A | G | G | G | G | no | polymorphic site |
| 4841 | ND2 | G | A |  |  |  | no | polymorphic site |
| 5100 | ND2 | C | T |  |  |  | no | polymorphic site |
| 5261 | ND2 | G |  |  | A |  | no | polymorphic site |
| 5351 | ND2 | A |  |  | G | G | no | polymorphic site |
| 5460 | ND2 | G |  |  | A | A | Ala > Thr | polymorphic site |
| 6179 | COI | G | A | A |  |  | no | polymorphic site |
| 6455 | COI | C |  |  | T | T | no | polymorphic site |
| 6671 | COI | T |  | C |  |  | no | polymorphic site |
| 6680 | COI | T |  |  | C | C | no | polymorphic site |
| 7028 | COI | C | T | T | T | T | no | polymorphic site |
| 7196 | COI | C | A | A |  |  | no | polymorphic site |
| 7598 | COII | G |  |  |  | A | Ala > Thr | polymorphic site |
| 7684 | COII | T |  |  | C | C | no | polymorphic site |
| 7853 | COII | G |  |  | A | A | Val > Ile | polymorphic site |
| 8572 | ATPase8 | G |  |  |  | A | no | polymorphic site |
| 8584 | ATPase6 | G | A | A |  |  | Ala > Thr | polymorphic site |
| 8684 | ATPase6 | C | T | T |  |  | Thr > Ile | polymorphic site |
| 8701 | ATPase6 | A | G | G | G | G | Thr > Ala | polymorphic site |
| 8860 | ATPase6 | A | G | G | G | G | Thr > Ala | polymorphic site |
| 9540 | COIII | T | C | C | C | C | no | polymorphic site |
| 9548 | COIII | G | A |  |  |  | no | polymorphic site |
| 9824 | COIII | T |  |  | C | C | no | polymorphic site |
| 10398 | ND3 | A | G | G | G | G | Thr > Ala | polymorphic site |
| 10400 | ND3 | C | T | T | T | T | no | polymorphic site |
| 10873 | ND4 | T | C | C | C | C | no | polymorphic site |
| 11255 | ND4 | T |  | C |  |  | Tyr > His | polymorphic site |
| 11719 | ND4 | G | A | A | A | A | no | polymorphic site |
| 12405 | ND5 | C |  |  | T | T | no | polymorphic site |
| 12705 | ND5 | C | T | T | T | T | no | polymorphic site |
| 12811 | ND5 | T |  |  | C | C | Tyr > His | polymorphic site |
| 13050 | ND5 | A |  | G |  |  | no | polymorphic site |
| 13401 | ND5 | T |  |  | C |  | no | polymorphic site |
| 13488 | ND5 | T | C |  |  |  | no | polymorphic site |
| 14256 | ND6 | T |  |  | C |  | no | no |
| 14470 | ND6 | T | C | C |  |  | no | polymorphic site |
| 14766 | Cytb | C | T | T | T | T | Thr > Ile | polymorphic site |
| 14783 | Cytb | T | C | C | C | C | no | polymorphic site |
| 14893 | Cytb | A |  |  |  | G | no | polymorphic site |
| 15043 | Cytb | G | A | A | A | A | no | polymorphic site |
| 15301 | Cytb | G | A | A | A | A | no | polymorphic site |
| 15326 | Cytb | A | G | G | G | G | Thr > Ala | polymorphic site |
| 15487 | Cytb | A | T | T |  |  | no | polymorphic site |
| 16129 | D-loop | G |  |  | A |  | no | polymorphic site |
| 16153 | D-loop | G |  | A |  |  | no | polymorphic site |
| 16184 | D-loop | C | T | T |  |  | no | polymorphic site |
| 16192 | D-loop | C |  |  | T |  | no | polymorphic site |
| 16223 | D-loop | C | T | T | T | T | no | polymorphic site |
| 16245 | D-loop | C | T |  |  |  | no | polymorphic site |
| 16278 | D-loop | C |  |  |  | T | no | polymorphic site |
| 16291 | D-loop | C |  |  | T |  | no | polymorphic site |
| 16293 | D-loop | A | G |  |  |  | no | polymorphic site |
| 16297 | D-loop | T |  |  | C | C | no | polymorphic site |
| 16298 | D-loop | T | C | C |  |  | no | polymorphic site |
| 16319 | D-loop | G | A | A |  |  | no | polymorphic site |
| 16519 | D-loop | T |  |  | C |  | no | polymorphic site |
| rCRS: revised Cambridge Reference Sequence | | | | |  |  |  |  |
| AA: Amino Acid | |  |  |  |  |  |  |  |
| ** databases: MITOMAP, mtDB, mtSNP and PhyloTree mt | | | | |  |  |  |  |
